# Subcutaneous Injection and Brush Application of Ovalbumin–Aluminum Salt Solution Induces Dermatitis-like Changes in Mice

**DOI:** 10.3390/jcm14051701

**Published:** 2025-03-03

**Authors:** Gabriel Siquier-Dameto, Ainhoa Iguaran-Pérez, Javier Gimeno-Beltrán, Gilberto Bellia, Andrea Maria Giori, Pere Boadas-Vaello, Enrique Verdú

**Affiliations:** 1 Research Group of Clinical Anatomy, Embryology and Neuroscience (NEOMA), Department of Medical Sciences, University of Girona, 17003 Girona, Spain; info@dametoclinics.com (G.S.-D.); ainho.30.perez@gmail.com (A.I.-P.); pere.boadas@udg.edu (P.B.-V.); 2 Dameto Clinics International, 07310 Campanet, Spain; 3 Servei de Patologia, Hospital del Mar, 08003 Barcelona, Spain; jgimenobeltran@psmar.cat; 4 IBSA Farmaceutici Italia, 26900 Lodi, Italy; gilberto.bellia@ibsa.it (G.B.);

**Keywords:** dermatitis, ovalbumin, alloknesis, mast cell, dermal collagen

## Abstract

**Background:** Intraperitoneal sensitization combined with topical and/or epicutaneous treatment using an ovalbumin (OVA)–aluminum salt solution (OVA-AL) represents a model for inducing atopic dermatitis (AD). However, the combination of sensitization with subcutaneous treatment and cutaneous application of OVA-AL via a brush has not been explored as a method for inducing AD. **Methods:** Adult mice were subcutaneously injected with OVA-AL following sensitization on days 0, 7, and 14 and were treated with OVA-AL via brush application to the dorsal skin fortnightly until days 35 and 49. Concomitant alloknesis and skin changes were assessed. Mice of the Balb/c and ICR-CD1 strains were treated with OVA-AL until day 35, with only the ICR-CD1 strain continuing treatment until day 49. Control animals received saline. At 35 and 49 days, dorsal skin was harvested and processed for histological analysis. **Results:** Mice treated with OVA-AL developed dry skin, with no scratching or alloknesis. Histological examination of dorsal skin revealed an increase in mast cells and collagen deposition. **Conclusions:** Dermatitis-like symptoms were observed in mice treated with OVA-AL using this administration method.

## 1. Introduction

Atopic dermatitis is an inflammatory skin disorder, with a prevalence of up to 20% in children and up to 3% in adults [1]. The key features of atopic dermatitis include pruritus, rashes, and lichenification in flexural areas, with a tendency to chronicity. A personal or family history of atopy—such as asthma, allergic rhinoconjunctivitis, or atopic dermatitis—is also common. Other frequent clinical manifestations include dry skin, facial pallor, keratosis pilaris, ichthyosis vulgaris, hyperlinearity of the palms and soles, white dermographism, conjunctivitis, keratoconus, elevated serum IgE, and immediate reactivity to skin tests [2]. Histologically, atopic dermatitis is classified as a spongiotic dermatitis progressing through three stages: (i) the *acute phase*, characterized by epidermal edema, which may or may not include prominent intercellular bridges or vesiculation, accompanied by normal ‘basket wave’ orthokeratosis; (ii) the *subacute phase*, which features acanthosis (epidermal thickening) with minimal edema and hyperkeratosis with or without parakeratosis (retention of nuclei in the stratum corneum); and (iii) the *chronic phase*, which exhibits marked acanthosis and hyperkeratosis typically without epidermal edema. In all phases of atopic dermatitis, perivascular chronic inflammation may be observed in the superficial dermis. In the acute phase, epidermal Langerhans cells’ micro-abscesses may also be present [3].

The current scientific literature suggests that the mechanisms underlying atopic dermatitis are multifactorial, involving environmental factors, alterations in the physical–chemical–immunological barrier of the skin, and dysbiosis of the cutaneous microbiota, in individuals genetically predisposed to the condition [4]. The stratum corneum plays a crucial role as a physical barrier against the entry of microorganisms. Mutations that induce functional changes in the filaggrin protein—a protein that binds to keratin fibers in the epithelial cells of the stratum corneum and helps maintain skin barrier integrity—have been associated with an increased risk of atopic dermatitis. Other genetic variants associated with atopic dermatitis include filaggrin-2, hornerin, loricrin, and SRP-3, all of which encode epidermal proteins important for skin barrier protection. Dysfunction in the skin’s protective barrier facilitates the entry of microorganisms such as Staphylococcus aureus, which penetrates the epidermal and dermal layers, triggering the skin’s immune response.

Regarding the cutaneous immune system, an increase in IgE and interleukins (e.g., IL4, IL13) has been observed in individuals with atopic dermatitis, as well as an overactivation of T helper (Th) lymphocytes (e.g., Th2, Th22, Th1, Th17), eosinophils cells, mast cells, macrophages, basophils, and dendritic cells (e.g., Langerhans cells), resulting in the production of chemical immune mediators such as interleukins (e.g., IL-1beta, IL6, IL8, IL10, IL21, IL22, IL25, IL31, IL33, IL36, IL37), chemokines (e.g., CCL18), cytokines (e.g., TNF-alpha), leukotriene C4, and interferon-gamma. Some of these chemical mediators trigger itching and scratching of the skin, further compromising the skin’s physical barrier and promoting the entry of microorganisms and fungi (e.g., *Staphylococcus aureus, Staphylococcus epidermidis, Candida albicans, Candida parapsilosis, Malassezia restricta, Malassezia. globosa, Malassezia. furfur, Malassezia. sympodialis, Alternaria, Aureobasidium, Aspergillus, Cladosporium* spp.). Furthermore, dendritic cells in the skin (e.g., Langerhans cells, dermal macrophages) recognize microorganisms through TLR receptors, and via their phagocytic capacity, become antigen-presenting cells. This process activates a broad repertoire of adaptive immune cells, particularly T and B lymphocytes, which in turn synthesize and secrete a large part of the aforementioned chemical mediators. Finally, various external factors and/or environmental factors—such as personal care products (e.g., soaps, detergents), changes in climate and ambient temperature, air pollution, ultraviolet radiation, consumption of allergenic foods/diets, lifestyle choices, stress, or socioeconomic factors— can exacerbate atopic dermatitis. These factors contribute to the dysbiosis of the cutaneous microbiota, leading to alterations in the epidermal barrier and the cutaneous immune system [1,4,5,6,7,8,9,10].

Chicken egg ovalbumin (OVA) has been administered epicutaneously and/or topically in animal models to induce changes like atopic dermatitis, being one of the classic experimental models of the development of atopic dermatitis [11]. According to the scientific literature, in murine models, OVA triggers a cutaneous inflammatory process that causes itching and skin lesions due to scratching, which exacerbate the inflammation of the skin, and all of which mimics the disease of atopic dermatitis [12,13,14,15,16,17,18]. This experimental OVA-induced model leads to significant infiltration of immune cells in the dermis, consisting of activated T cells, mast cells, and eosinophils [12,13,15,17,18,19,20,21,22,23,24], as well as an important response of Th2 lymphocytes with overexpression of interleukins (IL4, IL13, IL31, IL33, IL9, IL5), chemokines (CCL11, CCL26), interferon, and elevated IgE levels [11,12,13,17,21,22,25,26,27]. In addition, the skin of these OVA-treated animals shows symptoms of dryness, erythema, hemorrhage, excoriation, and edema [19,28,29], and the main histological changes induced by OVA administration are thickening of the treated skin areas [13,15,19,20,22,23,24,25,30]. These histological and biochemical/molecular changes develop and improve alloknesis [31], which has been defined as the itch evoked by a mechanical stimulus, that is, a touch-evoked itch [32,33].

Different strains of mice have been used to test the effect of OVA administration including Balb/c [12,13,14,15,17,18,20,22,23,24,26,29,34], C57BL/6 [14,20,21,31], SKH-1/Hr [18], SJL/J [12], 129Sv [14], and CD1 [35]. Most of the studies cited have used adult females between 4 and 10 weeks of age (preferably between 6 and 8 weeks old), except for very few studies that have used adult males between 6 and 8 weeks old [25,31]. Likewise, in most of the studies cited above, inbred mice (e.g., Balb/c, C57BL/6, SJL/J, 129Sv) have been used preferentially, and few studies have used outbred mice (e.g., SKH-1/Hr, ICR-CD1), but none of the previous studies make a comparison between inbred and outbred mice.

In the studies indicated above, epicutaneous administration of OVA has consisted of absorbent patches impregnated with the OVA solution, which are placed on the shaved and depilated skin of the back. In some studies, a small methacrylate box is even placed over the patch to prevent it from being removed by the animal [13,14,15,26,27,30]. Subcutaneous injection of OVA has been used as a sensitization method [36,37], but this route of OVA administration has not been previously explored as a method to induce skin inflammation and the development of dermatitis, and/or signs and symptoms like dermatitis. The subcutaneous route is one of those used to apply allergens that activate the immune system and trigger allergen immunotherapy [38]; it is also a route of administration of protein drugs and/or proteins [39], and in the context of animal welfare, subcutaneous administration is less aversive than other administration routes such as intramuscular [40,41].

All the findings described above suggest that mice treated with OVA constitute an experimental model of atopic-like dermatitis, developing most of the functional, histological, and molecular changes described in patients with atopic dermatitis [11,20,42]. The most relevant signs/symptoms in patients with atopic dermatitis are dry and inflamed skin, which triggers itching or pruritus, and this encourages skin scratching, which further exacerbates skin lesions [43,44,45,46,47]. The pathogenesis of atopic dermatitis combines dysfunction of the skin barrier (e.g., altered expression of the filaggrin gene, reduction in ceramides) that facilitates transcutaneous water loss and favors the entry of allergens, irritants, and microorganisms. Alteration of the skin barrier leads to inflammation of the skin with epidermal hyperplasia, and an increased number of mast cells, eosinophils, dendritic cells, and T lymphocytes. There is also immune dysregulation with activation of helper type 2 lymphocytes (Th2), which release cytokines (e.g., IL4, IL5, IL13, IL25, IL31). These cytokines released by Th2 lymphocytes but also by mast cells, eosinophils, and dendritic cells contribute to activating small diameter sensory neurons, generating sensations of itching and pain, via activation of TRP ion channels [44,45,48,49].

The objectives of the present work are first to study whether the subcutaneous and topical administration of the OVA–aluminum salt solution induces an increase in the number/density of mast cells in the skin of the back, as well as an increase in the thickness of the epidermis. It should be noted that the increase in the number of these cells together with the activation of Th2 lymphocytes and the secretion of inflammatory mediators, and the increase in the thickness of the epidermis, are the criteria for atopic dermatitis induced by OVA in experimental models [11]. The second objective is to determine if the administration of OVA triggers alloknesis. Alloknesis has been found in animals treated with OVA [31], but patients with atopic dermatitis do not show signs of pain other than itching and scratching. The third objective is studying these functional and histological changes indicated above in two strains of mice, one inbred (Balb/c) and another outbred (ICR-CD1). These three objectives have been assessed using subcutaneous injection together with topical application with a brush and variants of administration of OVA–aluminum salt solution, which have not previously been used.

It must be indicated that mice of both strains were subjected to repeated treatment with ovalbumin–aluminum salt solution (OVA group) or with saline solution (saline group), following the administration pattern described by Kim et al. [23] up to 35 days, and the pattern described by Bartnikas et al. [50] and Kopecki et al. [51] with modifications up to 49 days. At 21, 35, and 49 days of follow-up, alloknesis was assessed. On these days, the macroscopic appearance of the animal’s dorsal skin was also evaluated (dermatitis score).

## 2. Materials and Methods

### 2.1. Animals, Ethical Regulations, and Experimental Design

Adult female (Mus musculus) mice of 8 weeks of age (25–30 g of body weight) have been used in the present study. Mice of the Balb/c strain were purchased from Envigo (Barcelona, Spain), and mice of the Swiss ICR-CD1 strain were purchased from Janvier Laboratories (Le-Genest-Saint Isle, France). All in vivo experimental procedures were performed at the Bellvitge animal facility of the University of Barcelona, following the ARRIVE 2.0 guidelines, the ethical principles of the IASP for the evaluation of pain in conscious animals [42], and the Directive of the European Parliament and of the Council of 22 September 2010 (2010/63/UE), together with the approval of the Ethics Committee for Animal Experimentation of the University of Barcelona (CEEA; CEEA number 50/19; approved on 11 April 2019), and the Department of Agriculture, Live-stock, Fisheries, Food and the Natural Environment of the Generalitat de Catalunya, Generalitat de Catalunya (DAAM number 10672; approved on 22 November 2019).

Two regimens of repeated administration of OVA have been tested, one for 35 days [23] and another for 49 days [50,51]. At 21, 35, and 49 days of follow-up, alloknesis and the macroscopic appearance of the animal’s dorsal skin (dermatitis score) were assessed. On days 35 and 49, the animals were deeply anesthetized, intraventricularly perfused with histological fixative, and the dorsal skin was removed and processed via histological techniques.

### 2.2. Induction of Atopic Dermatitis

A solution of 0.5 g of ovalbumin (grade V; #A5503; Sigma-Aldrich-Merck, Darm-stadt, Germany) and 0.2 g of aluminum hydroxide hydrate (#A1577; Sigma-Aldrich-Merck, Darmstadt, Germany) were prepared in 500 mL of saline solution (0.9% Vitulia Physio-logical Serum, Barcelona, Spain) (OVA-AL solution) and another solution of 0.5 g of ovalbumin in 500 mL of saline solution (OVA solution) was also prepared. These solutions were filtered through 0.22 µm filters (syringe filters 0.22 NYL; #SFNY-122-100; Labbox, Barcelona, Spain) and dispensed into 50 mL tubes (#PTSP-E50-025; Labbox, Barcelona, Spain) and stored at 4 °C until use.

On days 0, 7, and 14, the animals received 0.2 mL of the OVA-AL solution intraperitoneally (i.p.). Likewise, on day 14 the animals were anesthetized with sodium pentobarbital (50 mg/kg; 10 mg/mL; i.p.), the skin of the back was shaved using an electric hair shaver, and then the shaved area was depilated using depilatory cream (Silky fresh sensitive skin; Veet, Reckitt Benckiser Healthcare, Hull, UK). The shaved and depilated area of dorsal skin was impregnated with OVA-AL solution using a Pelikan bristle brush (n° 10; Pelikan 721431; Hannover, Germany) (OVA group). Control animals received saline solution (i.p.) at 0, 7, and 14 days, and the shaved and depilated area of dorsal skin was impregnated with saline solution using another Pelikan bristle brush (Pelikan, Hannover, Germany) (saline group). Between days 15 and 21, daily the animals of the OVA group received 0.2 mL of OVA intraperitoneally, 0.2 mL of OVA-AL subcutaneously, and the dorsal skin was impregnated with OVA-AL by brush, while the animals of the saline group received 0.2 mL of saline solution intraperitoneally and subcutaneously, and the back was impregnated with saline solution with the corresponding brush. This regimen was repeated between days 28 to 35 and 42 to 49, leaving days 22 to 27 and 36 to 41 without administering any OVA solution or saline solution (weeks off).

It should be noted that the brush soaked in the OVA-AL solution, or the vehicle, was gently passed over the entire skin of the animal’s back, without applying pressure, but overcoming the resistance of the brush as it was moved across the shaved and depilated skin. The application time of the brush on the skin was 60 s (1 min), during which the brush was impregnated three times with the corresponding solutions, according to the experimental group. Once the solutions were applied with the brush, the dorsal skin of the animal was left uncovered and not occluded by any dressing meaning the skin remained exposed.

Regarding the experimental groups, two groups of animals were used in this study, (i) the saline group, treated with saline solution, and (ii) the OVA group, treated with ovalbumin (OVA) during sensitization with OVA-AL, and at different weeks (15–21, 28–35, 42–49) with OVA administered intraperitoneally.

### 2.3. Evaluation of Alloknesis and Cutaneous Alterations

At days 21, 35, and 49 after administering OVA or saline solutions, alloknesis and skin changes were evaluated. For this, each animal was placed in a conventional mouse cage (#1284L Eurostandard Type II L; Tecniplast, Buguggiate, Italy) for 10 min. After this time, alloknesis was determined using the method described by Akiyama et al. [52,53] with minor modifications. At 3 min intervals, each animal received 5 innocuous mechanical stimuli on the back, using the 2.83 von Frey filament that causes a force of 0.07 g. These stimuli were applied at 5 different points on the back. After applying the von Frey filament, the presence or absence of a positive response to stimulation was assessed, in the form of scratching the stimulated area and/or vocalization to the stimulus received (scratching and/or vocalization were considered positive responses). The final value of alloknesis was the total number of positive responses recorded at the five stimulation points on the back. For each positive response, 1 point was given, so the value of the alloknesis varied between 0 points (no response) to 5 points (maximum response).

After assessing alloknesis but keeping each mouse inside the cage, the degree of skin alterations was assessed using the dermatitis score described for rodents based on SCORAD. The SCORAD assessment is based on the degree of the development of erythema/hemorrhage, scarring/dryness, edema, and excoriation/erosion on the skin of the back was scored as 0 (none), 1 (mild), 2 (moderate), and 3 (severe). The sum of the individual scores was taken as the dermatitis score, which can range from a minimum score of 0 points (absence of dermatitis) to a maximum score of 12 points (very severe dermatitis) [54,55,56,57,58,59].

### 2.4. Histological Evaluation

At the end of follow-up and after performing all functional evaluations, animals were deeply anesthetized with sodium pentobarbital (90–100 mg/kg; 10 mg/mL; i.p.) and perfused intraventricularly with 4% paraformaldehyde solution in phosphate buffer saline (PBS; 0.1 M, pH = 7.4). Subsequently, the dorsal skin was removed and placed inside a jar filled with this same fixing solution for 25 days at 4 °C. Subsequently, the fixative solution was changed to a cryoprotective solution of 30% sucrose in PBS, for at least another 25–30 days at 4 °C.

Each dorsal skin sample was spread on a cork plate by means of needles placed at its edges. In this position, an approximately 2 cm^2^ square was cut out from the center of the extended skin, which was embedded with Tissue Freezing Medium (Ref: 0201-08-926; Leica, Barcelona), by using a special mold. Next, the mold with the skin was frozen by placing it inside a cryostat (CM1520, Leica, Barcelona, Spain) at −24 °C, obtaining a block of freezing medium with the skin inside. Next, the block was detached from the mold and placed on the cryostat stage, properly orienting the block with the skin, to obtain 20 µm-thick cross sections of the skin that were collected on pregelatinized slides.

Between 8 and 10 histological sections from each animal were stained with hematoxylin and eosin (H&E). To conduct this, first the sections were stained for 30 s with hematoxylin (#HEMA-HPS-500; Labbox, Barcelona, Spain), removing the excess staining with tap water. Next, a wash with 70% ethanol was performed for 5 min, and then the sections were stained with eosin (# EOYDE-S0D-500; Labbox, Barcelona, Spain) for two minutes. Finally, the slides with the histological sections already stained were dehydrated in increasing ethanol baths (70°, 96°, 100°, 100°) for 5 min/bath, xylene bath for 2 min, and finally the coverslip was mounted with DPX (#1.01.979.500; Merck, Darmstadt, Germany).

The images of the histological sections stained with H&E were used to calculate the thickness of the epidermis and dermis, also using the free software Image-J (version 2.14.0/1.54f). To achieve this, using the segmented lines command, 10 segmented lines were placed on the epidermis marked more intensely on the dermis, placing the origin at the edge of the epidermis, and the end at the base of the epidermis. Each unit of segmented lines gave a value of length (thickness) of the epidermis. The same was repeated for the dermis, placing the origin at the epidermis–dermis limit, and the end at the limit between the dermis and hypodermis (rich in adipose tissue). Thus, each unit of segmented lines gave a value of length (thickness) of the dermis. Finally, for each image, the final thickness of the epidermis and dermis was the average value of the 10 segmented lines units. In both the dermis and the epidermis, these 10 units of segmented lines were placed throughout the entire image of the histological section.

Another set of 8–10 histological sections of skin from each animal was used to stain with commercial Giemsa solution (#EOMB-MSD-1K0; Labbox, Barcelona, Spain) diluted in distilled water (1Giemsa: 4water, *v*/*v*) for 90 min. Subsequently, they were washed with 0.1% acetic acid and 96° ethanol for 15–20 s each wash. Next, three 2 min baths were carried out with methanol and finally another three 2 min baths with xylene. The coverslip was mounted with DPX. Giemsa staining allows the visualization of mast cells, in various tissues, including the skin [60,61,62]. The number of mast cells was manually counted from captured images of the histological sections stained with Giemsa, using Image-J (version 2.14.0/1.54f) software to avoid counting errors. Two independent investigators performed the counts in a blinded manner.

A separate set of 8–10 histological sections of skin from each animal was stained with Masson’s trichrome, which highlights collagen fibers (type I and IV) deposited in the dermis and epidermis [63,64,65,66]. A standard Masson trichrome kit (#TRIC kmA-100, Labbox, Barcelona, Spain) was used, and the manufacturer’s protocol was followed. After staining, the sections were rinsed with distilled water to remove excess dye and dehydrated in a series of 5 min baths with increasing concentrations of ethanol. Finally, after two minutes in a xylene bath, the coverslip was mounted with DPX. The thickness of the collagen fibers in the epidermis was measured from the images captured using the optical microscope and digital camera described above. The measurements were made using Image-J (version 2.14.0/1.54f) and the same method previously described for the histological sections stained with H&E. Two independent researchers assessed the collagen thickness using Masson’s trichrome stain.

### 2.5. Statistical Analysis

The functional, behavioral, and histological analyses were performed blindly, using a numerical code for each animal. Statistical comparison between the different experimental groups was made using Kruskal–Wallis and Mann–Whitney U non-parametric statistical tests. Results are shown as mean ± standard error of the mean (SEM), and in all statistical analyses the level of significance is *p* < 0.05. The statistical program GraphPad Prism 9.0 for Macintosh was used.

## 3. Results

### 3.1. Response of Alloknesis, Scratching, and Skin Lesions in Balb/c and ICR-CD1 Animals Treated with OVA–Aluminum Salt Solution

At days 21 and 35 of follow-up, none of the animals treated with OVA–aluminum salt (OVA group) solution showed positive responses to cutaneous stimulation with the von Frey filament of 2.83, suggesting that these animals treated with OVA via subcutaneous injection and cutaneous application with a brush did not show positive alloknesis responses. These animals did not present a scratching response to the mechanical stimulus, nor did they present audible vocalization responses.

On days 21 and 35 of the follow-up, the degree of skin injury on the back was evaluated using the dermatitis scale adapted for rodents [54,55,56,57,58,59]. At 21 days of follow-up, none of the animals from the different experimental groups showed signs of skin lesions. At 35 days of follow-up, dermatitis score values in Balb/c animals were 1.16 ± 0.21 and 0.18 ± 0.12 in mice treated with OVA and saline, respectively. In ICR-CD1 animals, these values were 0.33 ± 0.33 and 0 ± 0 in mice treated with OVA and saline, respectively. Significant differences (*p* < 0.05) were observed between saline-treated and OVA-treated animals in both mouse strains. Additionally significant differences (*p* < 0.05) were observed between OVA-treated Balb/c mice and OVA-treated ICR-CD1 mice.

**Figure 1:**
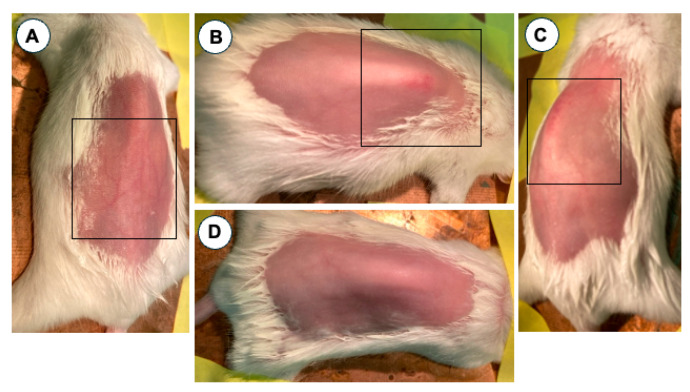
Images of the back of the mice treated with saline and OVA. At 35 days, animals treated with OVA do show dorsal lesions (**A**–**C**), while animals treated with saline do not show dorsal skin lesions (**D**). Areas with lesions are shown in the black box. (**A**,**B**,**D**) correspond to Balb/c mice and (**C**) to ICR-CD1 mice.

Animals with skin lesions mainly showed signs of dryness with erythema/edema and desquamation. Occasionally some of them showed a linear lesion of small partially confluent papules that formed a plaque. Both types of lesions are dermatitis-like typical (Figure 1). These results suggest that animals treated with OVA present mild skin lesions.

All these findings suggest that the subcutaneous injection of OVA together with the cutaneous impregnation of this solution with a brush triggers very mild lesions of the skin of the back that do not involve scratching the skin, nor a positive response to mechanical stimulation with the von Frey filament (alloknesis), in both strains of mice.

### 3.2. Histological Changes on the Skin of the Back in Balb/c and ICR-CD1 Animals Treated with OVA–Aluminum Salt Solution

At 35 days, the mast cell counts performed by independent researchers in the different experimental groups are presented in Table 1.

As shown in the table, there are no significant differences between the mast cell counts performed by the two independent researchers.

**Figure 2:**
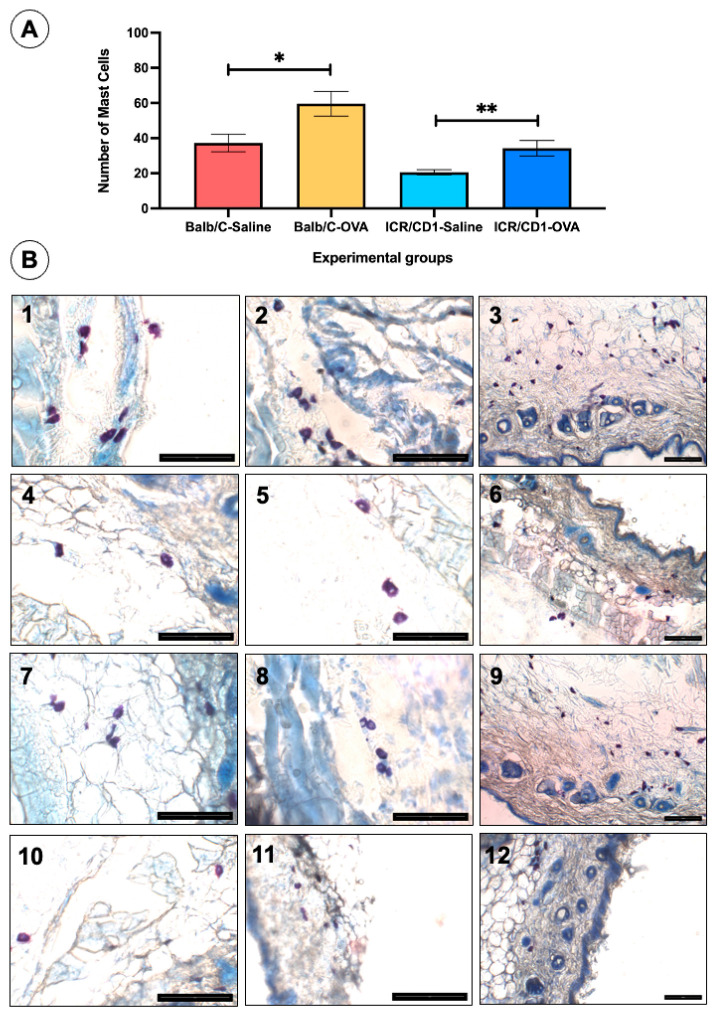
Mast cells result in the histological sections of the dorsal skin at 35 days in the different experimental groups. (**A**) The histogram shows a significant increase in the number of mast cells in the animals of the OVA-AL group compared to the control (saline) group in both strains of mice. Significant differences in mast cell numbers were observed between mouse strains, both saline- and OVA-treated (see text). (**B**) Panel of images showing mast cells from histological sections stained with Giemsa from the different experimental groups: Balb/c-OVA (1, 2, 3), Balb/c-Saline (4, 5, 6), ICR-CD1-OVA (7, 8, 9), ICR-CD1-Saline (10, 11, 12). Values are expressed as mean ± standard error of the mean (SEM). The number of animals per experimental group is 5 (*n* = 5). * *p* < 0.05 and ** *p* < 0.01 compared to the control group (saline). Scale bar = 100 µm for all panel images.

At 35 days of follow-up, the number of mast cells in the dermis of histological sections stained with Giemsa significantly increased in animals treated with OVA compared to those treated with saline in both strains of mice (Figure 2A). When comparing the number of mast cells between Balb/c and ICR-CD1 mice treated with OVA, the values were 59.60 ± 7.05 and 34.22 ± 4.41, respectively, with significant differences (*p* = 0.0028) observed between the two groups of animals. Similarly, the number of mast cells in saline-treated animals was 37.23 ± 5.06 and 20.52 ± 1.48 in mice of the Balb/c and ICR-CD1 strains, respectively, with significant differences (*p* = 0.0156) between the two strains. Mast cells stained with Giemsa appear violet in color with internal granules, though not all violet cells clearly show these granules. These cells were present in all histological sections from the different experimental groups (Figure 2B), with greater numbers observed in the groups treated with ovalbumin.

On the other hand, at 35 days of follow-up, the thickness of Masson’s trichome staining, evaluated by two independent researchers (researcher 1 and researcher 2) in the different groups, is shown in Table 2. No significant differences in collagen thickness were found between the two researchers who evaluated this parameter.

**Figure 3:**
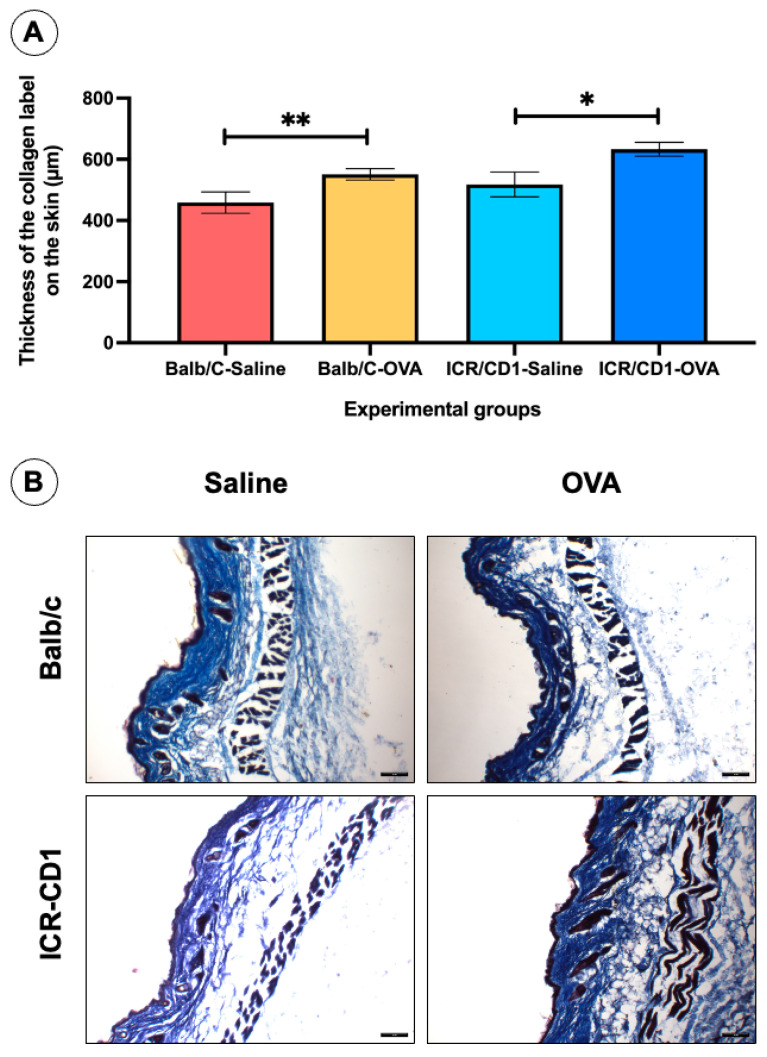
Results of the thickness of Masson’s trichrome labeling in histological sections of the dorsal skin at 35 days across the different experimental groups. Masson’s trichome labeling allows for the visualization of collagen fiber deposition in the skin. (**A**) The histogram shows that the thickness of this labeling is significantly greater in the OVA-treated experimental groups compared to the saline-treated control groups, in both strains of mice. (**B**) Panel of images of histological sections from animals in the different experimental groups stained with Masson’s trichrome, showing collagen deposition in both the epidermis and dermis. Values are presented as the mean ± standard error of the mean (SEM). The number of animals per experimental group is 5 (*n* = 5). * *p* < 0.05 and ** *p* < 0.01 compared to the control group (saline). Scale bar = 100 µm for all panel images.

The thickness of Masson’s trichrome staining in dorsal skin samples is significantly higher in animals treated with OVA compared to those treated with saline in both strains of mice (Figure 3A). Collagen deposition in the epidermis and dermis is visibly marked as an intense blue in the histological sections (Figure 3B). When comparing the thickness of the Masson’s trichomic staining in the histological sections of the animals treated with OVA from the Balb/c and ICR-CD1 strains, it was observed that the thickness was 458.8 ± 34.96 µm and 517.9 ± 40.50 µm, respectively, with no significant differences (*p* = 0.4023) between the two groups of mice.

Finally, at 35 days, in the histological sections of the dorsal skin stained with hematoxylin–eosin, it was revealed that the epidermal thickness was 27.28 ± 1.14 µm and 23.52 ± 0.92 µm in Balb/c animals treated with saline and OVA, respectively. Significant differences were observed between both experimental groups (*p* < 0.05). In the ICR-CD1 strain, the epidermal thickness was 26.51 ± 1.61 µm and 25.53 ± 1.12 µm in the animals treated with saline and OVA, respectively, with no significant differences observed between the two experimental groups. When comparing the epidermal thickness of OVA-treated animals, no significant differences were observed between Balb/c and ICR-CD1 strains (*p* = 0.2300). Regarding dermal thickness, in Balb/c animals, it was 190.37 ± 11.11 µm and 203.32 ± 14.37 µm in the histological sections of the dorsal skin from saline- and OVA-treated animals, respectively, with no significant differences between the two experimental groups (*p* > 0.05). Similarly, in ICR-CD1 animals, dermal thickness was 191.94 ± 12.05 µm and 172.77 ± 7.52 µm in saline- and OVA-treated animals, respectively, with no significant differences observed between the two groups (*p* > 0.05). When comparing the dermal thickness of OVA-treated animals, no significant differences were observed between Balb/c strains and ICR-CD1 strains (*p* = 0.5656).

These findings suggest that the subcutaneous injection of OVA solution together with the impregnation of the dorsal skin with this same solution using a brush increases the number of mast cells in the skin and the thickness of the epidermis compared to the saline-treated animals, in both strains of mice. The increase in cutaneous mast cells and epidermal thickness are indicators of atopic dermatitis in experimental models induced by OVA [11].

In summary, most of the parameters evaluated in OVA-treated mice are similar between both strains, with the exceptions of the dermatitis score and mast cells counts, which are higher in Balb/c mice than in ICR-CD1 mice (Table 3). Furthermore, when analyzing the functional, behavioral, and histological changes following the administration of OVA and saline, these changes are more pronounced after OVA treatment than after saline, a pattern that is consistent in both Balb/c and ICR-CD1 mice. These results suggest that an inbred strain does not offer significant advantages over an outbred strain, despite the higher cost of purchasing inbred mice. Consequently, the 49-day regimen was conducted using the outbred ICR-CD1 strain.

### 3.3. Functional and Histological Changes in ICR-CD1 Mice Treated with OVA–Aluminum Salt Solution

**Figure 4:**
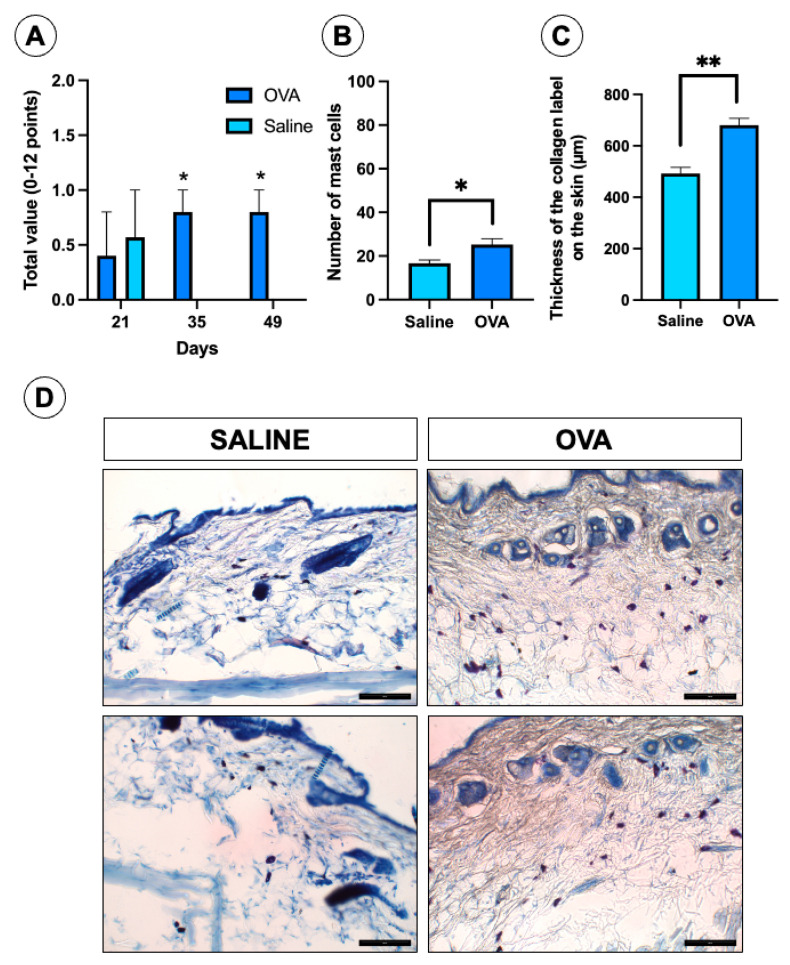
Results of ICR-CD1 mice subjected to OVA and saline treatments up to 49 days of follow-up. (**A**) Results of the assessment of the degree of skin alterations using the mouse dermatitis score in both experimental groups on the different days of follow-up. (**B**) Results of the mast cells count in the dorsal skin of both experimental groups at 49 days of follow-up. (**C**) Thickness of the marking of collagen fibers in the dorsal skin made by staining with Masson’s trichrome at 49 days of follow-up. (**D**) Panel of histological images of dorsal skin stained with Giemsa, from both experimental groups, in which blue-violet cells can be observed in the dermis, which correspond to mast cells. The quantity of these cells is greater in the images from the OVA group than in those from the saline group. Values are mean ± standard error of the mean (SEM). The number of animals per experimental group is 5 (*n* = 5). * *p* < 0.05 and ** *p* < 0.01 compared to the control group (saline). Scale bar = 100 µm.

In the pattern of repeated administration of OVA up to 49 days, none of the animals treated with OVA or saline showed signs of alloknesis when the skin of the back was stimulated with the dee von Frey 2.83 filament. In the assessment of skin changes, on day 21 of the follow-up, the animals of both experimental groups showed a similar score, but on days 35 and 49 of the follow-up only some animals treated with OVA showed signs of skin changes (Figure 4A). The main sign presented by the animals of both experimental groups was dry skin, which was observed in both groups at 21 days, but on the following days this sign of lesion skin only remained in animals treated with OVA.

On the other hand, at 49 days of follow-up the histological analysis of the dorsal skin showed a significant increase in the number of mast cells (Figure 4B,D) in the histological sections of the animals treated with OVA compared to those treated with saline. Likewise, the thickness of the labeling of collagen fibers from the dorsal skin, stained with Masson’s trichrome stain, was significantly higher in the histological sections of the animals treated with OVA compared to those treated with saline (Figure 4C).

Table 4 shows a summary of the main functional and histological changes observed at 35 and 49 days of follow-up in the ICR-CD1 animals treated with OVA or saline. The pattern of changes is similar between day 35 and 49 of the follow-up for most of the parameters evaluated. In general, the magnitude of change observed at day 49 of follow-up is slightly greater than that observed at day 35 of follow-up. Overall, by lengthening the exposure time to OVA, it causes more changes and of a slightly greater magnitude.

## 4. Discussion

The present study shows that animals treated with OVA present cutaneous signs of dry skin, which is not accompanied by alloknesis and skin scratching. Histologically, animals treated with OVA presented a greater number of mast cells in the dorsal skin, as well as a greater thickness of collagen, compared to animals treated with saline solution. These results were similar between the two mouse strains used. Comparatively, at 49 days the changes observed are slightly more intense than those observed at 35 days.

Previous studies have shown that mice sensitized and topically treated with OVA only showed redness, scaling, and dry skin compared to control animals [23,67,68,69]. There are studies that describe erythema and excoriation [31], skin inflammation and hypertrophic scars [70], erythema and thickening of the skin [51,71], edema, excoriation, and scaling [72], and bleeding, eczema, and dryness [73]. All these studies suggest that sensitization and topical treatment with OVA generate mild skin lesions such as erythema and dry skin, up to severe lesions with edema, excoriation, and desquamation, and even bleeding. In other words, the degree of skin injury is not uniform despite using the same procedure.

Several studies have shown that topical application of OVA causes itching or pruritus, which induces spontaneous skin scratching responses that contribute to the generation of skin lesions assessed by the dermatitis scale [31,74]. Based on the results observed in this study, it can be deduced that the level of itching caused by subcutaneous administration and cutaneous impregnation with a brush of OVA solution was not intense enough for the animals to have notorious and repeated scratching responses that would injure the dorsal skin. This deduction is also because the animals treated with OVA did not show an alloknesis response, although other authors did observe this response in animals also treated with OVA [31].

The topical application of inflammation-inducing agents generates itching, scratching, and alloknesis responses [53,75,76,77], which are caused by an increase in the number of mast cells [78,79,80,81,82] and eosinophils [83,84] into the inflamed skin. Eosinophils and mast cells in the skin are a characteristic feature of atopic dermatitis [85,86,87]. Both types of cells also increase in the skin of animals treated with OVA compared to animals treated with PBS or saline [15,16,17,88].

In the present study, a significant increase in mast cells was also observed in the skin of mice treated with OVA compared to mice treated with saline, even though no severe skin lesions were detected in these animals, and that all this apparently is not accompanied by itching and alloknesis. Various reasons can explain all this: the first reason may be that the animals do have itching, but in a very dorsal area and not very accessible to scratching with the hind legs; therefore, the skin lesions have been minimal, and this has not exacerbated the dermatitis. It is well known that scratching due to pruritus amplifies and perpetuates atopic dermatitis in both humans [89,90] and animal models [54,91,92]; however, mouse strains Balb/c and ICR-CD1 present fewer scratch responses to pruritic stimuli than other strains such as the NC/Nga and C3H/HeN under conditions of dermatitis [91]. Faced with pruritogenic stimuli administered subcutaneously, mice of the Balb/c strain were the ones that showed a significantly lower scratching response than 10 other unbred strains of mice, while the C57BL/6 strain is the one that showed very high signs of scratching [93]. Another reason may be related to the procedure of cutaneous application of ovalbumin (OVA) solution. Most studies use OVA application protocols using impregnated patches that adhere to the skin surface for a week at a time, allowing OVA to stay in contact with the skin longer. These studies describe skin scratch lesions, erythema, dry skin, and even bleeding [13,14,15,20,26,31,51,67,68,94,95]. In the present study, subcutaneous injection, and dorsal application of the OVA solution by brush is not enough to generate skin lesions as described above; however, this method of OVA application induces histological changes in the skin like those described when it is applied by skin patch. Tests prior to this study carried out by the research group confirm that the skin patches (Cosmopor E—7.2 × 5 cm; Hartmann S.A., Mataró, Spain) impregnated with OVA only last if the animals remain under the effects of anesthesia. By the time they wake up, they tend to remove the patch on each other’s back. Likewise, the use of dorsal tape does not make it easier for the patch to remain on the back, and on the contrary generates a greater stress response in the animals (unpublished results). It should be noted that there are researchers who have placed plastic protections (mini boxes) on the back of the animals to prevent them from removing the impregnated patch [96]. However, this procedure of the mini plastic boxes would not have been approved by any of the ethics committees to which the animal experimentation procedure that supports this work was presented.

OVA treatment also results in increased deposition of extracellular matrix components in the dermis, especially collagen. This increase in collagen deposition identified with Masson’s trichrome staining in animals treated with OVA has also been described by other authors at the level of the airways when there is inhalation of OVA as an asthma model [97,98] or rhinitis [99]. On the other hand, in skin biopsies of diabetic subjects there is an increase in mast cells and deposition of collagen fibers, with a positive relationship between both parameters [100]. In patients with breast cancer treated with radiotherapy, an increase in collagen deposition in the irradiated skin is observed, together with an increase in the number of mast cells [101]. Taken together, all these findings suggest that mast cells induce an increase in collagen deposition in the skin. There is evidence that mast cells promote fibroblast proliferation and collagen deposition, and that several mast cell factors (e.g., TGF-beta, TNF-alpha, histamine, tryptase, mast cell chymase) may be involved [101,102,103]. There are no previous studies that have evaluated the deposition of collagen in the skin of animals treated with OVA. The present study is the first to analyze collagen deposition in the skin of animals treated with OVA, which is also accompanied by an increase in skin mast cells. On the other hand, there is clinical evidence that suggests that an exaggerated deposit of skin collagen is accompanied by increased pruritus [104,105,106]. This excess collagen deposition in the dermis, which causes the skin to appear dry, is associated with increased interleukin-6 levels (IL6) [107] and TGF-β [108]. The TGF-β factor induces the secretion of IL33 by dermal dendritic cells, which in turn stimulates the sensory terminals responsible for the itch sensation [109]. IL6 is a factor that also induces itch [110]. Pruritic dermatitis conditions are also accompanied by increased IL6 levels [111,112,113]. Together, all these findings suggest that the increase in collagen in the skin of animals treated with OVA could be mediated by factors released by mast cells, and that all of this can potentially cause itching and skin scratching.

In the experimental model of epicutaneous patch sensitization, after shaving and waxing the skin on the back, a patch of various materials (e.g., sterile gauze, alginate dressing) measuring 1 × 1 cm was impregnated with ovalbumin solution and aluminum salts, and was placed on the back of the animal, securing it with a transparent bio-occlusive dressing or transparent adhesive tape [13,15,17,24,31,95]. In other cases, a Finn disk or chamber was used, held in place with transparent adhesive tape and soaked in the ovalbumin solution [27]. The patch and/or Finn disk were kept on the animal’s back for one week and then removed. After 2 weeks, another patch was placed on the same area of skin, and this was repeated three times. In this model, an increase in serum IgE has been observed, as well as an increase in chemical mediators in the skin including cytokines (e.g., IL4, IL5, Il10, IL13), chemokines (CCL17, CXCL9, CXCL10, CXCL11), interferon (e.g., IFN-gamma), and cellular elements (e.g., neutrophils, eosinophils, mast cells) has been observed [12,13,15,17,18,19,20,21,23,24,25,26,27,28,29,35,95]. Decreased levels of filaggrin have been described in the skin of animals treated with OVA [23,28]. An increase in the thickness of the epidermis and dermis has also been observed in this experimental model of atopic dermatitis [23,28]. In the present study, molecular changes in the skin of animals treated with ovalbumin and their respective controls were not evaluated, and plasma levels of IgE were not determined either. Thus, the lack of a cytokine profile may be one of the limitations of this work; therefore, future studies should be carried out to analyze the expression of IL4, IL13, and IL31 in the skin to confirm Th2 polarization, using the application method described in this work.

Nevertheless, compared with all these previous studies, the present study also observed an increase in mast cells in the skin treated with ovalbumin compared to control animals, as well as changes in the thickness of the epidermis and dermis. Although molecular changes in the skin after subcutaneous administration of ovalbumin cannot be affirmed in the present study, other previous studies have observed that repeated subcutaneous injection of OVA or OVA-AL causes an increase in eosinophils and neutrophils in the peritoneal region [114] and in the bronchoalveolar lavage fluid [115,116]. An increase in interleukins (IL4, IL13) is also observed in bronchoalveolar lavage fluid after repeated subcutaneous administration of OVA or OVA-AL [117]. These repeated subcutaneous injections of OVA or OVA-AL also induce an increase in plasma IgE and IgG levels [118,119]. Subcutaneous injection of OVA into the sole of the paw causes skin inflammation, with increased levels of interleukins (IL-1beta, IL6, IL13), cytokines (TNF-alpha), and chemokines (CCL11) in the skin of this region [120]. Subcutaneous injection of ovalbumin into the plantar skin of the mouse causes a cutaneous increase in proinflammatory factors (IL33, TNF-alpha, IL1-beta) generating mechanical hyperalgesia [121]. In a recent study, it was observed that regarding the thickness of the epidermis and dermis, the increase in skin thickness was greater in animals with subcutaneous injection of OVA-AL and less in animals with an OVA-AL patch. However, the level of IgE increased significantly in both animals treated with a patch and subcutaneous injection of OVA-AL. It should be noted that these authors reported issues with the group of mice treated with the OVA-AL patch, as the animals removed the patch, even when it was fixed with adhesive tape [122]. All of this evidence suggests that application of OVA or OVA-AL in a skin patch induces a local immune response at the patch site, whereas the subcutaneous injection of OVA or OVA-AL has both generalized immune effects and local immune effects at the treated skin. Furthermore, although not studied in detail in the present study, the immune responses of animals treated with ovalbumin using the methodology described in the present study may differ from the immune responses observed in epicutaneous sensitization with an ovalbumin-soaked patch. In this context, epicutaneous sensitization with application of a patch soaked in ovalbumin solution causes an increase in CD3+ cells [13,14,15,22], CD4+ cells [13,14,15,20,22,23,25,26,73], CD8+ cells [15], CD11+ cells [17,23,25,73], and CD45+ cells [13,14], as well as IL1 [13,23,35], IL4 [13,14,17,18,20,22,23,24,25,28,35,68], IL5 [13,14,24,28], IL6 [26], IL10 [17,28], IL9 [26], IL12 [22], IL13 [15,17,20,23,24,28,35], IL17 [18,26], IL31 [24,68], and interferon-gamma [13,14,17,26] in comparison to vehicle-treated animals. Following subcutaneous injection of ovalbumin, an increase in IL1 [120,121], IL6 [120], IL13 [120], and IL33 [121], as well as CD4+ cells and CD25+ cells [123], also were seen. All this experimental evidence suggests that the application of skin patches soaked in ovalbumin induces a more intense immune response, with a greater variety and quantity of interleukins and other immune mediators, as well as a higher number of immune cells, than the subcutaneous administration of ovalbumin.

On the other hand, cutaneous application of a microneedle patch loaded with an ovalbumin solution causes activation and proliferation of cytotoxic T cells and helper T cells, as well as an increase in IL2 and interferon gamma compared to a microneedle patch loaded with vehicle [124]. Likewise, in patches with microneedles soaked with ovalbumin it has also been observed that most cells in the lymph nodes are CD3+ cells (T lymphocyte cells; 63.7%), followed by B220+ cells (B lymphocyte cells; 32.4%), CD11b cells (2%), CD11c cells (1.1%), and natural killer cells (0.8%) [125]. The degree of immunogenicity observed with these patches with microneedles filled with ovalbumin is much higher than that observed with intramuscular injection of ovalbumin [126]. This route of cutaneous administration of ovalbumin using microneedle patches also causes higher levels of immunoglobulin G than subcutaneous administration of ovalbumin [127]. This evidence also suggests that the application of microneedle patches soaked with ovalbumin show a greater immunogenicity response than subcutaneous injection.

The administration of ovalbumin in different species of animals (e.g., rodents, lagomorphs) constitutes an experimental model of various pathologies such as allergic airway inflammation [128], allergic rhinitis [129,130], asthma [131,132,133], cough [134], hypersensitivity pneumonitis [135,136], anaphylactic shock [137,138], vasculitis [139], scleritis [140], allergic conjunctivitis [141,142], food allergy [143,144,145,146], autoimmune encephalomyelitis [147,148], eosinophilic otitis media [149,150], arthritis [151,152,153], polymyositis [154], and atopic dermatitis [13,15,17,18,28,31,155]. In all these pathologies, ovalbumin causes an increase in inflammatory/immunity processes. It is well known that ovalbumin (OVA) is not intrinsically immunogenic and therefore has to be injected in the presence of adjuvants (substances that increase the immunogenicity of an antigen), typically aluminum salt [156]. The administration of the OVA and aluminum salt solution (OVA-AL) triggers an activation of type 2 helper lymphocytes (Th2), an effect that is called sensitization. Further, immunization of OVA-AL to the sensitized animals synergizes the former immune response, and animals exhibited inflammation, infiltration of eosinophils, production of Th2 cytokines, and increase in serum IgE, changes observable in most of the previous pathologies induced by OVA-AL [157]. In the present work, we have used the administration of OVA-AL via subcutaneous injection and skin impregnation with a brush to generate skin changes like atopic dermatitis, with the future objective of using this procedure for inducing skin changes to apply potential therapies.

In the present study, animals treated with the ovalbumin dilution vehicle (saline solution) were used as the control group, consistent with previous studies [13,14,20,28,35,50,51]. However, another potential control group that could have provided additional insights would have been animals treated with ovalbumin without aluminum salts. This omission can be considered a limitation of the current study. Additionally, the lack of an epicutaneous positive control, i.e., animals treated with a patch soaked in ovalbumin and aluminum salts (the classic model of epicutaneous sensitization with ovalbumin) is another limitation of this work. Therefore, further studies are needed to compare the model described here with traditional skin patch-based models.

Despite these limitations, the experimental model described in this study was chosen because the animals removed the ovalbumin–aluminum salts-soaked patch. As previously mentioned, unpublished data indicated that animals removed the patch after awakening from anesthesia, even when the patches were secured with adhesive tape. This fixation method caused a significant stress response in the mice (unpublished results). Similar challenges with patch fixation have been reported by other researchers [122], as discussed earlier. To minimize these issues, alternative methods such as using mini plastic boxes on the dorsal side to prevent patch removal, preventing the animals from removing the patch [96], housing animals in isolation, or the combination of both could be employed.

Regarding housing, European and Spanish legislation on the care of experimental animals recommends group housing [158,159]. Consequently, the animals in this study were kept in groups of five per cage. However, this group housing arrangement required the removal of the patches soaked in OVA-AL, which led to the adoption of an alternative methodological approach in this study. The proposed model, although not without its advantages, disadvantages, and limitations, was chosen as the most suitable for the objectives of this work, as discussed in the preceding paragraphs.

As previously mentioned, the lack of alloknesis (i.e., scratching in response to mechanical stimuli in ovalbumin-sensitized skin that could cause itching) may be attributed to several factors: (i) the administration route of ovalbumin may not induce sufficient changes in the skin to trigger itch and scratching responses; (ii) the dorsal area of the sensitized skin may be inaccessible for scratching by the hind paws of the mice; and (iii) the strains of mice used may have a lower propensity for scratching behavior. In addition to these factors, the recent scientific literature on itch in atopic dermatitis suggests that itching shares similarities with pain, as both involve similar mechanisms. These include neuronal sensitization due to exposure to proinflammatory agents (e.g., cytokines, chemokines), activation of intracellular pathways in neurons common to both itch and pain (e.g., TRPV ion channels, voltage-dependent ion channels), and growth factors (e.g., NGF) that enhance both pain and itch in a feedback loop [160]. Furthermore, it has recently been observed that chronic scratching in individuals with atopic dermatitis or brachioradial pruritus triggers structural changes in peripheral nerve fibers, particularly an increase in the branching of nerve terminals in the epidermis [161]. The absence of alloknesis in the present study may also be due to a lower sensitization of peripheral nerve fibers, or to a reduced branching of nerve fibers innervating the skin, resulting from the minimal scratching behavior observed in Balb/c and ICR-CD1 animals. Immunohistochemical analysis of dorsal skin samples from OVA-treated mice, visualizing cutaneous nerve fibers immunoreactive to CGRP (calcitonin gene-related peptide) and/or to PGP9.5 (Protein Gene Product 9.5), could provide valuable insights into the terminal branching of these nerve fibers, which are described as nociceptive fibers. This analysis could help correlate potential structural changes in nociceptive fibers with the low or absent alloknesis responses observed [162,163].

Low sensitization and/or desensitization of the afferent nerve fibers through which sensory information related to itching and pain is transmitted may contribute to the absence of alloknesis. There are two primary pathways of pruritus (itching), each stimulated by different receptors: histamine receptors in the epidermis and cowhage receptors in the dermis. Action potentials are transmitted by mechanically insensitive C fibers and polymodal C fibers, respectively, to secondary neurons in the dorsal horn [164].

At the molecular level, there are three G-protein-coupled histaminergic receptors (H1, H2, H3) for histamine. These receptors activate phospholipase C, phospholipase A2, and transient receptor potential vanilloid 1 (TPRV1), which increases intracellular calcium levels in skin-specific dorsal root ganglion sensory neurons. In contrast, cowhage cleaves the extracellular domain of protease-activated receptor 2 (PAR2), activating phospholipase C, TRPV1, and transient receptor potential ankyrin 1 (TRPA1), resulting in membrane depolarization [164].

The receptor common to both itch and pain pathways is TRPV1, as it is activated by both histamine and cowhage stimuli. It is known that persistent stimulation of the TRPV1 receptor by chemical stimuli such as inflammatory mediators (e.g., interleukins, cytokines) triggers receptor activation, which at the neuronal level leads to a decrease in excitability and results in complete insensitivity to subsequent stimuli, a process known as desensitization [165]. Activation of the TRPV1 receptor by inflammatory mediators causes calcium ions to enter neurons. This increase in intracellular calcium activates various calcium-dependent intracellular cascades leading to the dephosphorylation of the receptor, which in turn triggers receptor desensitization [166,167]. As a result, the nerve fibers no longer respond to pruritogenic stimuli, resulting in the desensitization of the pruritogenic sensory receptors.

Recently, an increase in TRPV1 expression in skin nerve fibers has been observed in atopic dermatitis induced with trinitrochlorobenzene in Nc/Nga Mice [168]. In the ovalbumin-induced atopic dermatitis model, intrathecal injection of IL-31 has been shown to evoke pruritus in mice, with the IL-31 receptor co-localizing in TRPV1-positive DRG neurons [169].

These findings suggest that the TRPV1 receptor is expressed in experimental models of atopic dermatitis, and that its activation by interleukins triggers pruritus (itching). Furthermore, experimental evidence indicates that TRPV1 receptor expression is lower in the afferent nociceptive fibers of Balb/c mice compared to C57BL/6 mice [170]. Overall, nerve fiber desensitization could be attributed to the lower expression of TRPV1 receptors, which varies depending on the animal strain, leading to a loss of alloknesis.

## Figures and Tables

**Table 1:** Mast cell count results by two independent researchers.

Groups	Research 1	Research 2	
Balb/C-Saline	37.64 ± 6.05	36.82 ± 8.42	*p* = 0.7599
Balb/C-OVA	55.40 ± 9.13	63.80 ± 11.03	*p* = 0.5421
ICR/CD1-Saline	21.33 ± 2.30	19.75 ± 1.96	*p* = 0.6325
ICR/CD1-OVA	33.11 ± 6.56	35.33 ± 6.29	*p* = 0.6823

**Table 2:** Thickness of Masson’s trichrome staining results by two independent researchers.

Groups	Research 1	Research 2	
Balb/C-Saline	546.6 ± 30.57	554.9 ± 23.9	0.7283
Balb/C-OVA	458.7 ± 54.42	458.9 ± 46.16	0.6058
ICR/CD1-Saline	623.9 ± 34.40	642.7 ± 30.70	0.9430
ICR/CD1-OVA	517.7 ± 60.11	518.1 ± 58.45	0.7984

**Table 3:** Summary of the main results obtained after 35 days of monitoring in the two strains of mice treated with ovalbumin (OVA).

	Balb/c Mice	ICR-CD1 Mice
Alloknesis	None	None
Dermatitis score	++	+/none
Mast cells	+++	++
Thickness of epidermis	=	=
Thickness of dermis	=	=
Thickness of Masson’s trichomic labeling	=	=

NOTE: “=”: equal; “+”: very slight increase; “++”: slight increase; “+++”: moderate increase.

**Table 4:** Functional and histological changes observed in ICR-CD1 animals at 35 and 49 days of follow-up.

Parameter	35 Days of Follow-Up	49 Days of Follow-Up
Alloknesis	No	No
Skin lesion	Yes (dryness)	Yes (dryness)
Mast cells in dorsal skin	OVA > saline	OVA > saline
Collagen staining on dorsal skin (Masson’s trichome stain)	OVA > saline	OVA > saline

NOTE: “>”: value greater than.

## Data Availability

All data generated or analyzed during this study are included in this published article.

## Abbreviations

The following abbreviations are used in this manuscript:

129Sv	129 parental strain mice
AD	Atopic dermatitis
ARRIVE guides	Animal research reporting of in vivo experiments guides
Balb/c	Bagg albino-c mice
C3H/HeN	C3H/Heston mice
C57BL/6	C57BLACK6 mice
CCL11	C-C motif chemokine 11
CCL26CGRP	C-C motif chemokine 26Calcitonine gene-related peptide
DPX	Dibutylphthalate polystyrene xylene
H&E	Hematoxylin and eosin
IASP	International Association for the Study of Pain
ICR-CD1 or CD1	Institute of Cancer Research (ICR)-CD1 mice
IgE	Immunoglobulin E
IL13	Interleukin-13
IL25	Interleukin-25
IL31	Interleukin-31
IL33	Interleukin-33
IL4	Interleukin-4
IL5	Interleukin-5
IL6	Interleukin-6
IL9	Interleukin-9
NC/Nga	NC/Nagoya university mice
OVA	Ovalbumin
OVA-AL	Ovalbumin–aluminum salt solution
PBSPGP9.5	Phosphate buffer salineProtein Gene Product 9.5
SEM	Standard Error of the Mean
SJL/J	Swiss Jim Lambert mice
SKH-1/Hr	SKH1 Hairless mice
TGF-β	Transforming Growth Factor beta
Th2	Helper type 2 lymphocyte
TRPTRPV1	Transient Receptor Potential ion channel Transient receptor potential vanilloid 1
